# Beliefs and Attitudes toward Vegetarian Lifestyle across Generations

**DOI:** 10.3390/nu2050523

**Published:** 2010-05-17

**Authors:** Peter Pribis, Rose C Pencak, Tevni Grajales

**Affiliations:** 1 Department of Nutrition and Wellness, Andrews University, 8475 University Boulevard–Marsh Hall 313, Berrien Springs, MI 49104-0210, USA; Email: pencakr@andrews.edu; 2 Department of Educational & Counseling Psychology, Andrews University, 4195 Administration Drive–Bell Hall 159, Berrien Springs, MI 49104-0104, USA; Email: tevni@andrews.edu

**Keywords:** vegetarians, vegetarian diets, dietary patterns, Adventists, attitude, beliefs

## Abstract

The objective of the study was to examine whether reasons to adopt vegetarian lifestyle differ significantly among generations. Using a Food Frequency Questionnaire (FFQ), we identified that 4% of the participants were vegans, 25% lacto-ovo-vegetarians, 4% pesco-vegetarians and 67% non-vegetarian. Younger people significantly agreed more with the moral reason and with the environmental reason. People ages 41–60 significantly agreed more with the health reason. There are significant differences across generations as to why people choose to live a vegetarian lifestyle.

## 1. Introduction

A vegetarian diet is defined as a diet “consisting wholly of vegetables, fruits, grains, nuts, and sometimes eggs or dairy products” [1]. There are many variations of vegetarian diets. Semi-vegetarians avoid meat, poultry and fish most of the time. Pesco-vegetarians avoid meat and poultry but eat fish. Lacto-ovo-vegetarians avoid all meat, fish, and poultry but do eat milk, cheese, yogurt, other dairy products and eggs. Vegans avoid in their diet all products of animal origin [2]. 

Different vegetarian diet variations are chosen for different reasons depending on age, gender, religion, educational level and overall perceived health beliefs. A study publish in 1992 found that the highest number of vegetarians, 46 percent, chose a vegetarian diet for health reasons, 15 percent chose to be a vegetarian for animal rights reasons, 12 percent for friend/family influence, 5 percent for ethical reasons, 4 percent for environmental issues and 18 percent indicated other reasons [3].

A study conducted in the Netherlands researched the attitudes towards food and health among adults. The results showed that vegetarians had smaller households, higher education levels, higher socioeconomic status, lived in more urbanized residential areas; tended to agree that product information, specialty shops, health and ecological products, and social relationships were important, and were more ‘health-occupied’ than the meat eaters [4]. A study conducted in the UK examined the attitudes toward following a meat, vegetarian or vegan diet and the role of ambivalence (emotions) on these attitudes. The results indicated that people tend to have most positive beliefs and attitudes towards their own diets, and most negative beliefs and attitudes towards diets that differ from their own [5].

There has been an increase in the interest and popularity of the vegetarian lifestyle overtime. According to a research conducted by the Vegetarian Resource Group, in 1994 approximately 1% of U.S. population could be considered vegetarian; 2.5% in 2000; 2.8% in 2003 and 2.3%, which represents about 7 million people, by 2006 [6]. A poll conducted by the same group in 2008 discovered that about 6.7% of people always order a vegetarian dish when eating out (up from 5.5% in 1999) [7]. The proportion of young people who are vegetarian is still higher (6–11%), with similar levels of vegetarian teenagers being reported in both the United Kingdom and Australia [8,9,10]. 

Although there has been increased interest in the vegetarian lifestyle overtime, it is not clear what the main reasons are as to why people adopt this lifestyle. The focus in this report is to examine the beliefs and attitudes towards a vegetarian lifestyle across generations and to report on a theoretical model of the relationships between attitude, beliefs, knowledge and misconception concerning vegetarian lifestyles.

## 2. Experimental Section

### 2.1. Recruitment of Subjects

This cross-sectional, observational study was completed at Andrews University which is a Seventh-day Adventist (SDA) institution of higher learning. SDA represent a unique population known for their wide range of dietary habits. This conservative religious group prohibits the use of alcohol, tobacco, and pork and recommends that members adhere to lacto-ovo-vegetarian diet [11,12]. The study was approved by the University’s Institutional Review Board (IRB protocol # 07-122). Participants were drawn from a large undergraduate introductory-level nutrition class that is open to students from all academic directions. Students were recruited by the instructor and assured that anonymity and confidentiality would be maintained. Participation in the study was voluntary. Those who choose to participate received ten bonus points which were counted toward their final grade. Data collection took place over the Thanksgiving holiday in 2007. Students were asked to recruit their parents and grandparents for participation in this survey. 

### 2.2. Assessment of Food Intake and Attitudes toward Vegetarian Lifestyle

Each participant was asked to complete a four-page Lifestyle Practices Survey which consisted of four parts. Section one had 11 basic census questions (gender, ethnicity, marital status, education, occupation, age, *etc.*). In section two a 29-item Food Frequency Questionnaire (FFQ) was used to accurately ascertain the vegetarian status of the participants. In section three, questions addressed the use of herbs and supplements. In section four participants were asked to describe which lifestyle they practice (non-vegetarian, pesco-vegetarian, lacto-ovo-vegetarian, or vegan). Using a Likert Scale from 1 to 5 (strongly disagree [1]–agree[2]–no opinion[3]–agree[4]–strongly agree[5]) participants answered questions concerning their attitudes, beliefs, knowledge, and misconceptions about vegetarian lifestyles (Table 1).

**Table 1:** Selected questions used to assess nutritional knowledge, health food beliefs, attitudes toward vegetarian lifestyle and nutritional misconceptions.

**Nutritional Knowledge**
It is healthy to eat a handful of nuts daily
Flaxseeds and fish are good sources of omega-3 fatty acids
There are water-soluble and fat-soluble vitamins
**Health Food Beliefs**
“Organic foods” are better for your health because they contain more vitamins, minerals and other important nutrients
“Health foods” give people more energy than “regular foods”
**Attitudes Toward Vegetarian Lifestyle**
Vegan lifestyle is extreme
Being vegetarian is too complicated in today’s society
Vegetarian lifestyle is the healthiest option we have
Being vegetarian is cool
To be vegetarian you must have a strong personality
**Nutritional Misconceptions**
Today foods have so many vitamins added that people don’t have to worry about their nutrition
As long as appropriate weight is maintained a person doesn’t have to worry about nutrition

### 2.3. Statistical Analysis

Data were analyzed using One-Way-ANOVA, Pearson correlation and Structural Equation Modeling (SEM) [13] techniques with SPSS (version 15.0) and AMOS 7.0 statistical software. One-Way-ANOVA was used to test comparison between age groups. Descriptive data was tested for normality. Pearson correlations were examined to check for the internal validity of the data. In the development of the lifestyle questionnaire cluster analysis was used to group the questions into four separate areas labeled: knowledge about nutrition; beliefs in health foods; attitudes about vegetarian lifestyle; and nutritional misconceptions. *P* ≤ 0.05 was considered statistically significant.

## 3. Results and Discussion

### 3.1. Sample Size and Characteristics

Overall there were 609 participants who completed the survey. Descriptive data are shown in Table 2. Out of the 609 participants, 215 (35%) were male and 394 (65%) were female. The mean age was 32.0 years for males and 30.6 years for females. Body Mass Index (BMI) was calculated for all participants. The mean BMI was 25.0 for males and 24.4 for females. Using the Food Frequency Questionnaire (FFQ), we identified that 4% of the participants were vegans, 25% lacto-ovo vegetarians, 4% pesco-vegetarians and 67% non-vegetarians. 

**Table 2:** Selected characteristics of the study population (*n* = 609).

	Males	Females
Gender (%, *n*)	35.3 (215)	64.7 (394)
Age (years; mean, SD)	32.0 (17.4)	30.6 (17.3)
BMI (kg/m^2^; mean, SD)	25.0 (4.8)	24.4 (5.7)
Seventh-day Adventist (%, *n*)	74.9 (161)	81.7 (322)
**Ethnicity (%, *n*)**		
Caucasian	49.3 (106)	51.0 (201)
African American	18.6 (40)	17.3 (68)
Hispanic	12.1 (26)	11.4 (45)
Asian	9.3 (20)	6.3 (25)
**Marital Status (%, *n*)**		
Single	62.3 (134)	65.2 (257)
Married	29.8 (64)	24.4 (96)
**Vegetarian Status (%, *n*)**		
Non-vegetarian	74.4 (160)	63.5 (250)
Lacto-ovo-vegetarian	20.9 (45)	27.4 (108)
Pesco-vegetarian	2.3 (5)	4.3 (17)
Vegan	2.3 (5)	4.8 (19)
SD stands for Standard deviation; BMI stands for Body Mass Index		
The percentages in the columns do not add up to 100% because of missing data.		

### 3.2. Reasons for Vegetarian Lifestyle

**Figure 1:**
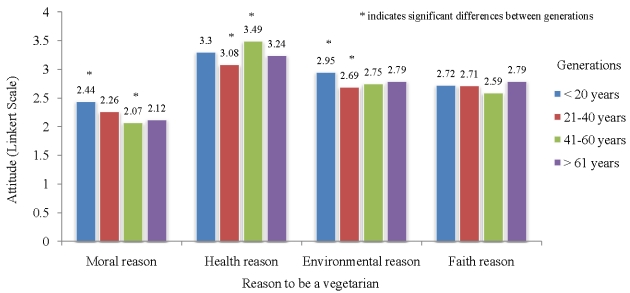
Distribution of attitudes concerning different reasons to be vegetarian across generations.

The lifespan of a generation is not clearly defined. Depending on the cultural norms for marrying age it is generally 20 to 30 years per generation. The age distribution of the population did create four clusters of similar age groups, however, with not very clearly defined beginnings and ends. We have done several statistical analyses defining generation between 20 to 25 years. They all provided somewhat similar results, therefore we are reporting the results using following generational categories: 11–20 years, 21–40 years, 41–60 years, and 61 and older. We asked four questions concerning reasons why they choose a vegetarian lifestyle—the moral reason (it is wrong to kill animals), the health reason (vegetarians live longer and are less sick), the environmental reason (vegetarian lifestyle is much more protective against the environment) and (because 80% of our respondents were Seventh-day Adventists) the faith reason (being vegetarian is part of Adventist lifestyle). The results (Figure 1) showed that the younger people (11–20 years) significantly agreed more with the moral reason (p = 0.003). People ages 41–60 significantly agreed more with the health reason (p = 0.010). Finally, younger people (11–20 years) also significantly agreed more with the environmental reason (p = 0.025). There were no significant differences concerning the faith reason (p = 0.715).

### 3.3. Verification of Vegetarian Status

Previous studies raised concerns that self-defined vegetarian status can be an unreliable indicator of true dietary preferences [14,15,16,17]. Table 3 represents the self-defined *versus* verified vegetarian status of the subjects. The bolded numbers represent those that defined their dietary preferences correctly. In order to make the self-identification process easier when asking participants what lifestyle they follow, the different vegetarian lifestyles were defined, e.g., vegan was defined as—eats vegetables, fruits, legumes, grains; lacto-ovo-vegetarian as - eats dairy products, eggs, vegetables, fruits, legumes, grains, *etc.* The results vary greatly according to the group. In non-vegetarians 97% of females and males identified themselves correctly, in pesco-vegetarians only 32% of females and 17% of males identified themselves correctly. In lacto-ovo-vegetarians 82% of females and 78% of males identified themselves correctly, and in vegans 48% of females and 57% males identified themselves correctly. The data for vegans however need to be interpreted carefully because of small numbers. 

**Table 3:** Self-defined *vs.* Verified Vegetarian Status by Gender (*n* = 600).

Self-defined status	Verified status							
	Vegan		Lacto-ovo-vegetarian		Pesco-vegetarian		Non-vegetarian	
	Male	Female	Male	Female	Male	Female	Male	Female
Non-vegetarian (*n*, %)	0 (0)	0 (0)	4 (2.8)	5 (2.2)	1 (0.7)	2 (0.9)	**137 (96.5)**	**216 (96.9)**
Pesco-vegetarian (*n*, %)	0 (0)	0 (0)	0 (0)	5 (14.7)	**2 (16.7)**	**11 (32.4)**	10 (83.3)	18 (52.9)
Lacto-ovo-vegetarian (*n*, %)	1 (2.0)	3 (3.0)	**39 (78.0)**	**83 (82.2)**	1 (2.0)	4 (4.0)	9 (18.0)	11 (10.9)
Vegan (*n*, %)	**4 (57.1)**	**15 (48.4)**	2 (28.6)	15 (48.4)	1 (14.3)	0 (0)	0 (0)	1 (3.2)
The bold underlined numbers indicate numbers and percentages of participants who were able correctly identify their vegetarian status								

### 3.4. Theoretical Model of the Relationship between Attitudes, Beliefs, Knowledge and Misconceptions Concerning Vegetarian Lifestyles

**Figure 2:**
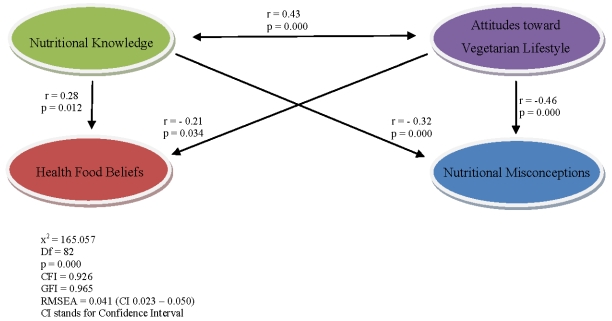
Structural Equation Modeling testing a theoretical model of the relationship between attitudes, beliefs, knowledge and misconceptions concerning vegetarian lifestyles.

This study examined the way underlying health concepts could explain why people chose vegetarian lifestyle using the SEM statistical method. SEM is a powerful multivariate statistical method being used in social sciences, and with increasing frequency in health behavior research. SEM examines underlying relationships among variables in the model and helps to explain social or behavioral phenomena [13]. Our model (Figure 2) was constituted by four sets of concepts: the *Attitudes toward vegetarian lifestyle*, *Nutritional knowledge*, *Nutritional misconceptions* and *Health food beliefs*. How are *Attitudes toward vegetarian lifestyle* related to *Nutritional knowledge*, *Health food beliefs* and *Nutritional misconceptions*? 

The hypothesized model was assessed by AMOS version 7.0 using the maximum likelihood method. The model was evaluated by four fit measures: **a**, the chi square **b**, the Comparative Fit Index (CFI) **c**, the Good-of-Fit-Index (GFI) and **d**, the Root Mean Square of Approximation (RMSEA). The results for three out of the four indices support the proposed model. The chi square had a value of 165.057 (Df = 82, n = 609), p=0.000, indicating a non-acceptable match between the proposed model and the observed data. However due to the size of the sample additional fitted indices were considered. The CFI = 0.926, GFI = 0.965, both of them indicating an excellent fit of the model. The RMSEA measures the discrepancy between the sample coefficients and the population coefficients equals 0.041 (confidence interval 0.023 – 0.050) indicating an acceptable fitting [18].

Findings support model that suggests that the *Attitudes toward vegetarian lifestyle* are significantly correlated with *Nutritional knowledge* (r = 0.43, p = 0.000) and have negative effect on *Health food beliefs* (r = −0.21, p = 0.034) and *Nutritional misconceptions* (r = −0.46, p = 0.000). The observed effect between *Nutritional knowledge* and *Attitudes toward vegetarian lifestyle* (r = 0.43, p = 0.000) is bilateral suggesting that these variables influence each other. Increased nutritional knowledge might lead to positive attitude toward vegetarian lifestyle, and vice versa vegetarian lifestyle may promote increase in nutritional knowledge. Further, *Nutritional knowledge* has negative effect on *Nutritional misconceptions* (r = −0.32, p = 0.000) and positive effect on *Health food beliefs* (r = 0.28, p = 0.012). The model seems to indicate that in our population positive attitude toward vegetarian lifestyle is knowledge base instead of just being nurtured by some traditional nutritional beliefs or misconceptions. Positive attitudes toward vegetarian lifestyle contribute to the reduction of nutritional misconception and non scientific beliefs about health foods.

The data presented in this paper indicate that there are significant differences across generations as to why people choose to live a vegetarian lifestyle. Young people under the age of 20 seem to choose vegetarian lifestyle for moral and environmental reasons, while the middle age group of people between ages 41 to 60 seem to choose this lifestyle for health reasons. This trend seems understandable, given the wealth of publications documenting the health benefits of vegetarian and low-meat diets [11,19,20,21,22]. For younger people health issues are not priority, however as they age this increases in importance as shown by the data. The health reason to be vegetarian in our population produced the strongest attitudes on the Likert scale, confirming previous finding that Adventist traditionally chose vegetarian lifestyle for health reasons. 

### 3.5. Study Limitations

Several potential limitations to this study should be considered. This is a population-based cross-sectional study, which included both genders and all age groups. The study was conducted on a campus of a American private university which may limit the generalizibility of the results. Although the sample size of the population was large enough, some groups such as the over sixty or vegans were underrepresented so the results should be interpreted with caution. Although SEM is a sophisticated analytic tool for testing theoretical models in behavioral or social science, the analyses are correlational which makes it difficult to establish causality. Because the isolation of variables in the model are impossible, all models must be looked at only as estimation of reality [23].

## 4. Conclusions

There are significant differences across some generations as to why people choose to live a vegetarian lifestyle. Younger people seem to be motivated by moral and environmental reasons, while those who are middle-aged seem to be motivated by health reasons. In our study, the non-vegetarians and lacto-ovo-vegetarians had the least difficulty correctly identify their vegetarian status. In our population the positive attitude toward vegetarian lifestyle is more knowledge based (supported by scientific information and facts) instead of just being fostered by some traditional nutritional beliefs or misconceptions (based on popular ideas and folkloristic practices).

## References

[1] (2003). Merriam-Webster’s Collegiate Dictionary.

[2] Messina V., Messina M. (1996). The Vegetarian Way.

[3] Sabaté J. (2001). Vegetarian Nutrition.

[4] Hoek A.C., Luning P.A., Stafleu A., de Graaf C. (2004). Food-related lifestyle and health attitudes of Dutch vegetarians, non-vegetarian consumers of meat substitutes, and meat consumers. Appetite.

[5] Povey R., Wellens B., Conner M. (2001). Attitudes towards following meat, vegetarian and vegan diets: an examination of the role of ambivalence. Appetite.

[6] Stahler C. (2006). How many adults are vegetarians?. Vegetarian J..

[7] Vegetarian Resource Group (2008). How Many People Order Vegetarian Meals When Eating Out?. Vegetarian J..

[8] Vegetarian Resource Group (2005). How Many Youth Are Vegetarians? How Many Kids Don’t Eat Meat?. Vegetarian J..

[9] Food Standards Agency National Diet and Nutrition Survey: Young people 4-18 yrs, Preliminary Results, Released June 2000. The Vegetarian Society U.K.: Statistics: Children/Young People.

[10] Worsley A., Skrzypiec G. (1998). Teenage vegetarianism: Prevalence, social and cognitive context. Appetite.

[11] Beeson W.L., Mills P.K., Phillips R.L., Andress M., Fraser G.E. (1989). Chronic disease among Seventh-day Adventists, a low-risk group. Cancer.

[12] Fraser G.E., Sabate J., Beeson W.L., Strahan T.M. (1992). A possible Protective Effect of nuts consumption on risk of coronary heart disease. Arch. Intern. Med..

[13] Buhi E.R., Goodson P., Neilands T.B. (2007). Structural Equation Modeling: A primer for Health Behavior Researchers. A J. Health Behav..

[14] Janelle K.C., Barr S.I. (1995). Nutrient intakes and eating behavior scores of vegetarian and nonvegetarian women. J. Am. Diet Assoc..

[15] Barr S.I., Chapman G.E. (2002). Perception and practices of self-defined current vegetarians, former vegetarian, and non-vegetarian women. J. Am. Diet Assoc..

[16] Haddad E.H., Tanzman J.S. (2003). What do vegetarians in the United States eat?. Am. J. Clin. Nutr..

[17] White R., Frank E. (1994). Health effects and prevalence of vegetarianism. West J. Med..

[18] Kline R.B. (2005). Principles and Practice of Structural Equation Modeling (Methodology in the Social Sciences).

[19] Chang-Claude J., Frentzel-Beyne R. (1993). Dietary and lifestyle determinants of mortality among German vegetarians. Int. J. Epidemiol..

[20] Fraser G.E., Shavlik D.J. (2001). Ten years of life: is it a matter of choice?. Arch. Intern. Med..

[21] Knutsen S.F. (1994). Lifestyle and the use of health services. A J. Clin. Nutr..

[22] Singh P.N., Sabate J., Fraser G.E. (2003). Does low meat consumption increase life expectancy in humans?. A J. Clin. Nutr..

[23] Bollen K.A. (1989). Structural Equations with Latent Variables.

